# Transitioning from Laboratory-Developed Tests to a Single Commercial Reagent Kit in a National Newborn Screening Program: Impact on Analytical Performance and Harmonization

**DOI:** 10.3390/ijns12020041

**Published:** 2026-06-09

**Authors:** Rachel S. Carling, Zoe J. Barclay, Sophie C. Ward, Marie Appleton, Robert Barski, Harry Benn, Kelly Chambers, Paul Coakley, Helena Kemp, Nicola Crabbe, Sarah Dowden, Toby Greenfield, Sarah L. Hogg, Saima Hussein, Rhiannon Marr, Oliver Parkes, Darren Powell, Tejswurree Ramgoolam, Joshua Ssali, Nazia Taj, Katherine Wright, Teresa H. Y. Wu, James R. Bonham

**Affiliations:** 1 Biochemical Sciences, Synnovis, Guys & St Thomas’ NHSFT, London SE1 7EH, UK; zoe.barclay@synnovis.co.uk (Z.J.B.); sophie.ward@synnovis.co.uk (S.C.W.); 2 GKT School of Medical Education, Kings College London, London WC2R 2LS, UK; 3 Newcastle Metabolic and Newborn Screening, Royal Victoria Infirmary, Newcastle upon Tyne NE1 4LP, UK; marie.appleton1@nhs.net (M.A.); kelly.chambers5@nhs.net (K.C.); 4 Biochemical Genetics, St James’s University Hospital, Leeds LS9 7TF, UK; robert.barski@nhs.net (R.B.); saima.hussein@nhs.net (S.H.); 5 Newborn Screening, South Yorkshire and Bassetlaw Pathology, Sheffield Teaching Hospitals NHSFT, Sheffield S10 2TH, UK; harry.benn@nhs.net (H.B.); katherine.wright2@nhs.net (K.W.); 6 Alder Hey Children’s NHS(FT), Liverpool L12 2AP, UK; paul.coakley@alderhey.nhs.uk (P.C.); darren.powell@alderhey.nhs.uk (D.P.); 7 Newborn Screening and Metabolic Biochemistry Department, North Bristol NHS Trust, Southmead Hospital, Bristol BS10 5NB, UK; helena.kemp@nbt.nhs.uk (H.K.); nicola.crabbe@nbt.nhs.uk (N.C.); 8 Newborn Screening and Biochemical Genetics, Birmingham Women’s and Children’s Hospitals NHSFT, Birmingham B4 6NH, UK; sarah.dowden@nhs.net; 9 Blood Sciences, Portsmouth Hospitals Trust, Portsmouth PO6 3LY, UK; toby.greenfield@nhs.net; 10 Biochemical Genetics Unit, Cambridge University Hospitals NHS FT, Cambridge CB2 0QQ, UK; sarah.hogg@nhs.net; 11 Clinical Biochemistry, Oxford University Hospitals NHS(FT), Oxford OX9 3DU, UK; rhiannon.marr@ouh.nhs.uk (R.M.); nazia.taj@ouh.nhs.uk (N.T.); 12 Willink Biochemical Genetics Laboratory, Genomic Medicine, Manchester University NHSFT, Manchester M13 9WL, UK; oliver.parkes@mft.nhs.uk (O.P.); hoiyee.wu@mft.nhs.uk (T.H.Y.W.); 13 Department of Chemical Pathology, Great Ormond Street Hospital for Children, London WC1N 3JH, UK; tejswurree.ramgoolam@gosh.nhs.uk; 14 South West Thames Newborn Screening Laboratory, South West London Pathology, Epsom & St Helier Hospitals, Carshalton SM5 1AA, UK; j.ssali1@nhs.net; 15 Sheffield Children’s NHS(FT), Sheffield S10 2TH, UK; j.bonham@nhs.net

**Keywords:** harmonization, standardization, succinylacetone, newborn screening, neonatal screening, mass spectrometry, laboratory-developed test, inherited metabolic disease

## Abstract

Newborn screening in England is a national program with laboratories adhering to common screening algorithms. Until recently, screening for inherited metabolic disorders was provided by ten laboratories using laboratory-developed tests (LDTs) and three using commercial assays: harmonization of results proved challenging. Introduction of hereditary tyrosinemia type 1 screening meant LDTs required modification to include the measurement of succinylacetone, and subsequent re-validation. This provided an opportunity to implement a single commercial reagent kit in all laboratories. It was anticipated that this would improve analytical performance and harmonization. This study aimed to determine whether these goals were achieved. Verification across the 13 laboratories revealed that the commercial kit reduced inter-laboratory variation for all analytes demonstrating improved harmonization. However, this was achieved by applying instrument-specific correction factors to all analytes, the magnitude of which were significant, indicating a lack of standardization. Performance of succinylacetone was limited by instrument-dependent background interference from the methionine stable isotope label, underscoring the need to establish evidence-based screening cut-off values (COV) rather than adopting published thresholds. This study emphasizes the need for traceable reference materials to improve laboratory quality and the value of screening outcome data.

## 1. Introduction

At the outset of this work newborn screening (NBS) in England was delivered as part of a national program with all babies being screened for nine disorders: Congenital Hypothyroidism, Cystic Fibrosis, Sickle Cell Disease, Phenylketonuria (PKU), Medium-Chain Acyl-CoA Dehydrogenase Deficiency (MCADD), Isovaleric Acidemia (IVA), Glutaric Aciduria Type 1 (GA1), Maple Syrup Urine Disease (MSUD) and pyridoxine unresponsive Homocystinuria (HCU). The latter six inherited metabolic diseases were detected using multiplexed flow injection analysis tandem mass spectrometry (FIA-MS/MS), with ten laboratories using laboratory-developed tests (LDT), and three laboratories using commercial reagent kits. All laboratories adhered to national screening algorithms based on single analyte screening cut-off values (COV), with disease status being suggested by a marker analyte concentration above the COV. The English screening program is widely recognized as being evidence-based, rigorously quality-assured, and among the most integrated and well-structured screening systems in the world. It has a focus on continuous quality improvement initiatives [1,2,3,4,5,6,7], several of which have aimed to improve harmonization between laboratories [2,4].

In parallel to these recent harmonization initiatives, the transition to the In Vitro Diagnostic Regulation EU 2017/746 (IVDR) has been underway in Europe. This has stimulated discussion around the potential consequences for screening labs using LDTs when equivalent European conformity (CE) marked tests are available on the market, with traceability, documentation, performance evaluation and risk management likely to be directly impacted. Whilst the UK is not regulated by the IVDR, the Medicines & Healthcare products Regulatory Agency (MHRA) is expected to publish its new regulatory framework in 2026, and the UK will then be in an extended transition period until June 2030. Irrespective of any regulatory requirements, the disadvantages of using LDTs in newborn screening are becoming increasingly apparent, including their impact on harmonization and standardization both within a given program [2,4] and between different programs [8,9,10], as well as the requirement for operator expertise to develop and maintain these methods. Commercial kits are considered to provide operational advantages such as ease of use, simplified reagent management, and supplier support. Perhaps most importantly, they also provide the potential for screening programs to be expanded by simply modifying the mass spectrometer (MS) acquisition parameters.

In 2024, the English government approved the introduction of screening for hereditary tyrosinemia type 1 (HT1; OMIM #276700) and specified that screening should be performed using succinylacetone (SA) in a dried blood spot (DBS). Irrespective of sample matrix or methodology, measurement of SA is acknowledged to be challenging [11,12,13]. Extraction of SA from a DBS requires an acidic solution which means that methanol, which is used to extract amino acids and acylcarnitines in the LDTs, is unsuitable. Early methods [14] accommodated this by introducing a two-step process: extracting the amino acids and acylcarnitines from the DBS and removing the supernatant, then adding hydrazine to the residual DBS punch to extract and derivatize the SA. Further optimization of the methods enabled the simultaneous extraction of amino acids, acylcarnitines and SA through two different approaches. The first extracted and derivatized amino acids and acylcarnitines using butyl esterification and then extracted and derivatized SA from the residual DBS punch using hydrazine, before combining the extracts and measuring all analytes by FIA-MS/MS [15,16]. The second approach extracted all analytes from the DBS in a single step via the sequential addition of methanol and hydrazine containing solutions [17]. In addition to these LDTs, at least four commercial reagent kits are now available.

It is evident from previous reports [18,19] that there are significant differences between methods used to measure SA, with LDTs showing more variability than commercial kits, irrespective of whether they are derivatized or non-derivatized methods. The Neobase™ 2 (NB2) method group displays the smallest inter-laboratory variation and has the highest number of users. Under-recovery is an issue for all methods: a proficiency sample enriched to a concentration of 50 µmol/L had a mean measured concentration of 23.4 µmol/L (approximate range 10 to 100) [18]. As expected, this absence of harmonization and/or standardization is reflected in the range of SA COVs used in screening programs throughout the world. A worldwide survey of NBS programs [19] found screening COVs ranged from 0.3 to 7.0 µmol/L (median 1.5, *n* = 31), whilst data from the Newborn Screening Quality Assurance Program (NSQAP) in 2022 reported the ranges in U.S and international laboratories were 0.4 to 6.1 µmol/L (median 2.0, *n* = 40) and 0.4 to 8.0 µmol/L (median 1.8, *n* = 112) respectively. The higher screening COVs tend to be associated with LDTs, irrespective of whether they are derivatized or non-derivatized, with less variation evident within a kit method group; for example, NB2 COVs ranged from 0.6 to 2.0 μmol/L (*n* = 10), whereas LDTs ranged from 1.0 to 7.0 μmol/L (*n* = 9). In the absence of harmonization, comparison of COVs has limited utility at best, and at worst could be misleading.

Taking the above factors into consideration, the implementation of HT1 screening in England was aligned with the transition to a single commercial reagent kit, NB2. Although there is relatively limited information available on the analytical performance of this method within a single laboratory [20,21], and even less between laboratories [18], it was anticipated that use of a single reagent kit would reduce inter-laboratory variation and improve harmonization across the English laboratories.

This study aimed to verify analytical performance of NB2 across the 13 laboratories, compare population distributions obtained using NB2 with historic LDT data and determine whether the transition to a single commercial kit improved harmonization.

## 2. Materials and Methods

Neobase™ 2 non-derivatized MSMS installation kit, non-derivatized assay solutions, succinylacetone solution, frozen controls (low, high), frozen internal standards, and multi-level DBS were obtained from Revvity (Turku, Finland). Third-party internal quality control (IQC) materials, MassCheck^®^ Amino Acids, and Acylcarnitines EXTENDED dried blood spot control levels I, II and III were obtained from Chromsystems (Munich, Germany). All materials were used as specified by the manufacturer.

All 13 English NBS laboratories participated in this study and each laboratory verified the method on two instruments; 4 TQD; 8 Xevo TQD and 5 TQ-S micro (Waters Corporation, Milford, MA, USA); 3 API 4500; 1 API 5000; 1 API 5500; 2 API 6500 (Sciex, Framingham, MA, USA); and 2 Shimadzu 8050 (Shimadzu Corporation, Kyoto, Japan). Initial optimization of the NB2 method on each instrument was performed by Revvity Field Service Engineers. Mass positions, mass resolution and compound dependent parameters, e.g., cone voltage, collision energy, and dwell time, were optimized by direct infusion of a tune solution prepared from the frozen internal standards. MStune and acquisition files, autosampler configurations and inlet method files, and post-analytical data processing files were created for the following analytes and their corresponding stable isotope label (SIL): phenylalanine (PHE); tyrosine (TYR); leucine (LEU); methionine (MET); isovalerylcarnitine (C5); glutarylcarnitine (C5DC); octanoylcarnitine (C8); decanoylcarnitine (C10); and SA. All samples were prepared as instructed by the manufacturer. Each of the nine analytes was quantified from the ratio of analyte to internal standard (IS) signal response, multiplied by IS concentration. A default relative response factor (RRF) for each analyte was included in the data processing method. RRF are analyte specific multiplication factors applied to the final measured result for each analyte. Preliminary assessment of intra-batch imprecision and linearity was performed by replicate analysis of frozen kit controls (*n* = 30, two levels) and multi-level DBS controls (*n* = 4, six levels) respectively. Results for the nine analytes were compared with the manufacturer-assigned values.

Subsequent optimization of the RRFs for each instrument was performed by each laboratory. Replicates (*n* = 12) of low and high kit control materials were analyzed on each of three different days. The mean measured results for each material were compared with the manufacturer-assigned values for the nine analytes. The final RRF for each analyte was the mean of the RRF from the replicates of the low and high control material.

Verification of the method on each instrument was performed by all laboratories according to a common protocol. Linearity was verified using the six DBS multi-level materials with acceptable performance defined as R^2^ > 0.99 (*n* = 6). The lower limit of quantitation (LLOQ) was not formally assessed due to the lack of matrix matched reference materials, the complexity associated with accurately preparing DBS samples and the absence of clinical impact at this level. Instead, replicates (*n* = 12) of endogenous material (DBS multi-level 1) were analyzed and imprecision at this level was compared with standard acceptance criterion (coefficient of variation (CV) ≤ 20%, *n* = 5 from at least three different batches). Recovery was assessed by replicate analysis (*n* = 3) of the six DBS multi-level materials, after the measured value had been corrected for endogenous concentration (back calculated). Carry-over was assessed by injection of an extracted blank (filter paper only) directly after injecting extracted kit high IQC material. Replicates of the blank (*n* = 6) were injected followed by six alternate replicates of a high IQC and blank, followed by a further six blanks: BBBBBBCBCBCBCBCBCBBBBBB. Acceptable performance was defined as signal intensity for the blank ≤ 20% of the signal intensity in DBS multi-level L1.

Intra-laboratory variation was assessed by replicate analysis (*n* = 20) of three third party IQC materials, on each instrument. Inter-laboratory variation was assessed by replicate analysis (*n* = 5) of three third-party IQC materials on each of five different days, on all instruments. Acceptable performance for inter-laboratory variation was defined as CV ≤ 15%, except at the LLOQ where CV ≤ 20% was accepted. Inter-lot variation was determined using the same 5 × 5 format but with the IS kit lot becoming the variable, rather than instrument or laboratory. The percentage relative difference between the mean of each lot of IS was determined rather than the mean bias to account for the fact that it is not known which lot represents the ‘true’ value.

On-board stability was assessed by repeat measurement of residual DBS specimens (*n* = 82) and IQC materials that had been left on the autosampler at 10 °C overnight. Samples were reconstituted with 100 µL of (a) 50% methanol and (b) 50% acetonitrile and mixed prior to re-injection. Stability was defined as no significant constant or proportional bias, and plate means within ±10%. The degree of interference from the MET SIL to endogenous SA was estimated by monitoring the signal intensity ratio of SA to MET SIL in extraction working solution on representative instruments, with extended longitudinal monitoring performed at laboratory 11.

To evaluate whether the clinical COV currently specified in the national screening algorithm for the LDTs would remain applicable for NB2, each laboratory analyzed approximately *n* = 1000 residual NBS specimens on each instrument (final data set *n* = 22,049). Population data was reviewed by instrument, laboratory and collectively. Statistical analysis included determination of mean, median, and population centiles (P1, P10, P90, P99) for each analyte and comparison with historic data (*n* = 131,128) obtained using the LDTs between October and December 2024. The median absolute deviation (MAD) was calculated to provide a robust measure of statistical dispersion around the median; however, emphasis was placed on the P90 as this was the centile closest to the clinical COV at which the data set size would provide a statistically meaningful confidence interval across all 26 instruments. The analytical COV is set approximately 20% below the screening COV. Samples which exceed the analytical COV are re-tested in duplicate and if the mean of the three results exceeds the screening COV, the baby is classified as screen-positive and referred for confirmatory testing. Inter-laboratory variability was assessed using the standard deviation (SD) of laboratory level MADs. The ratio of between-laboratory SDs of MADs (NB2/LDT) was used to assess relative variability with ratios < 1.0 indicating reduced inter-laboratory variation for NB2. This dispersion ratio was also calculated for the SDs of laboratory-level P90 and period centered P99; inclusion of the latter enables assessment of inter-laboratory variability in the extreme upper tail. The separation margin between the 99th centile and the screening COV was calculated to indicate discrimination. Rain shower plots were produced with the 90th centile ± 95% confidence interval (CI) shown for each data set.

Permission for the use of residual NBS specimens following anonymization was obtained from the NHS Population Screening Research, Innovation and Development Advisory Committee (ANNB-2425-007). Analysis was limited to those specimens which had already completed the NBS process, and for which sufficient material would remain for a further 3.2 mm sub-punch to be taken.

## 3. Results

### 3.1. Analytical Performance

The optimized analyte RRFs for each instrument are summarized in Supplementary Data, Table S1. RRFs ranged from 0.72 to 1.61 across the nine analytes. For C5 and C8 carnitine, RRFs were between 0.8 and 1.2 across all instruments. The widest range of RRFs was seen for C10 carnitine (0.98–1.61), SA (0.72–1.34) and C5DC carnitine (0.77–1.55). For C10 carnitine, 15 instruments had RRFs ≥ 1.2, and for SA, seven instruments had RRFs which were either <0.8 or >1.2. A review of the instrument model showed that the Waters TQ-S Micro was associated with the highest mean RRFs for PHE (1.13), TYR (1.14) and LEU (1.09) compared to the other models, although the significance of this finding is unclear. Laboratory 7 stood out as anomalous with all RRFs > 1.0 and a mean across both instruments of 1.16 (range 1.01–1.55). This pattern is quite striking: there is not a single RRF below 1.0 across the entire laboratory, which is unusual given that RRFs could logically be expected to be scattered either side of 1.0.

Overall, 13% of RRFs were adjusting the measured result by more than 20%. The underlying principle of stable isotope dilution mass spectrometry (SID-MS) is that the presence of an isotopically enriched version of the analyte of interest will compensate for any variability due to extraction efficiency, ionization efficiency and matrix suppression/enhancement because it will behave identically to the native analyte. The magnitude and range of the RRFs reported here are at odds with this and appear more indicative of fundamental differences between instruments, particularly given the commonality of stable isotopes, reagents and other materials.

Linearity was demonstrated to 1434, 1304, 1260, 842, 42, 20, 7, 7 and 183 µmol/L for PHE, LEU, TYR, MET, C8, C5, C5DC, C10 carnitines and SA respectively for most instruments, consistent with the manufacturer’s claims. The exceptions to this were: Sciex 4500 (3A, 4B) which went non-linear between 717 and 1434 µmol/L for PHE, and 652 and 1304 µmol/L for LEU; Sciex 4500 (3A) which went non-linear between 21 and 42 µmol/L for C8 carnitine; and Sciex 5000 (9B) which went non-linear between 717 and 1434 µmol/L for PHE, 21 and 42 µmol/L for C8, and 3.6 and 7.1 µmol/L for C10 carnitine. As these regions of non-linearity are well removed from the COVs typically used by NBS programs around the world, this is not problematic per se; however, it highlights the need for laboratories to establish linearity for each instrument to ensure the screening outcome data is meaningful. Importantly, it also emphasizes that SID-MS-based screening methods may be unsuitable for monitoring dietary therapy in patients with phenylketonuria. At a PHE concentration of 600 µmol/L, a key decision point in terms of patient management [22], instrument 3A and 4B would report concentrations of approximately 503 and 442 µmol/L respectively.

The mean recovery from all instruments was 100 ± 20% for all analytes except for succinylacetone (53%, range 31–72). See Table S2 in Supplementary Data. Also notable was the consistent over-recovery of C10 (112%, range 97–130) on all but two instruments. Recovery was determined relative to the Revvity multi-level DBS materials which is an acknowledged limitation as these are not higher-order standards but have manufacturer-assigned values; however, given that the mean RRF for C10 was 1.22 (Table S1), this points to a property of the kit materials, either the frozen controls or the C10 SIL, rather than an instrument-specific analytical issue, and underlines the absence of higher-order traceable reference materials against which to independently verify these values.

Carry-over was negligible for all analytes on all instruments. Mean intra- and inter-laboratory variation is summarized in Table 1.

Mean inter-lot variation across all laboratories for all analytes in each of the three levels of third-party IQC materials was ≤15% and directly comparable with the manufacturers’ claims. The exception to this was SA with 35.2, 18.0 and 25.3% CV for levels 1, 2 and 3 respectively. The mean bias (range) between lots was within ± 3% (−1.7 to 2.6). Results are summarized in Table S3. These data were used to establish an evidence-based process for verifying a new lot of IS prior to routine use and specifying the acceptance criteria.

On-board stability was demonstrated for samples reconstituted in 100 µL of either 50% methanol or 50% acetonitrile. When reconstituted in 50% methanol, plate means were within ±10% for all analytes except SA (20.1%). When reconstituted in 50% acetonitrile, plate means were within ±10% for all analytes except C10 (+13%) and SA (34%). As it is acknowledged that endogenous SA is below the LLOQ, the nominal difference between plate means was not considered to be of clinical significance and importantly, at the higher concentrations seen in IQC materials, mean bias was within ±10% for all analytes, and the bias across the different analytes was random, not consistent, irrespective of which solvent was used for reconstitution.

### 3.2. Lower Limit of Quantitation and the Impact of Isotopic Interference on Succinylacetone Quantitation

Replicate measurements of DBS multi-level 1 material on each instrument met the imprecision criteria for LLOQ for all analytes except for SA. SA failed the imprecision criteria on Waters TQD (10B, 12B), Waters Xevo TQD (1A, 1B, 2A, 8A, 13B) and Shimadzu 8050 (9A), and failed bias criteria on all instruments. Endogenous concentrations of SA in DBS specimens from healthy neonates are essentially undetectable by this methodology; the signal measured in DBS multi-level 1 reflects cross contribution from the MET SIL rather than true endogenous SA. The MET SIL is ^2^H_3_-methionine (transition 153.1 > 107.1), which contains a naturally abundant +2 isotopic contribution (155.1 > 109.1) of approximately 4% due to ^34^S. This transition is identical to that of the stable SA derivative, 3-(5-methyl-1H-pyrazol-3-yl) propanoic acid (MPP), formed during reaction with hydrazine and measured as a surrogate marker for SA. Consequently, background signal attributable to the MET SIL contributes directly to the apparent endogenous SA concentration. At higher concentrations, SA can be quantified with acceptable precision despite the background contribution from the MET SIL. This was demonstrated using the third-party level two QC material (nominal concentration 2.2 µmol/L) where mean intra-laboratory imprecision was 11.6% and inter-laboratory variation was 9.6%. These findings indicate that the LLOQ for SA lies between the instrument specific background signal and that of the level two IQC material. Importantly, the magnitude of this background is instrument-specific, varying from approximately 0.2 µmol/L on a Waters Xevo TQD, to 0.7 µmol/L on a Sciex 6500. This signal does not reflect the theoretical 4% isotopic contribution expected from natural ^34^S abundance, as the manufacturer ‘de-tunes’ each MS to minimize the interference.

**Figure 1:**
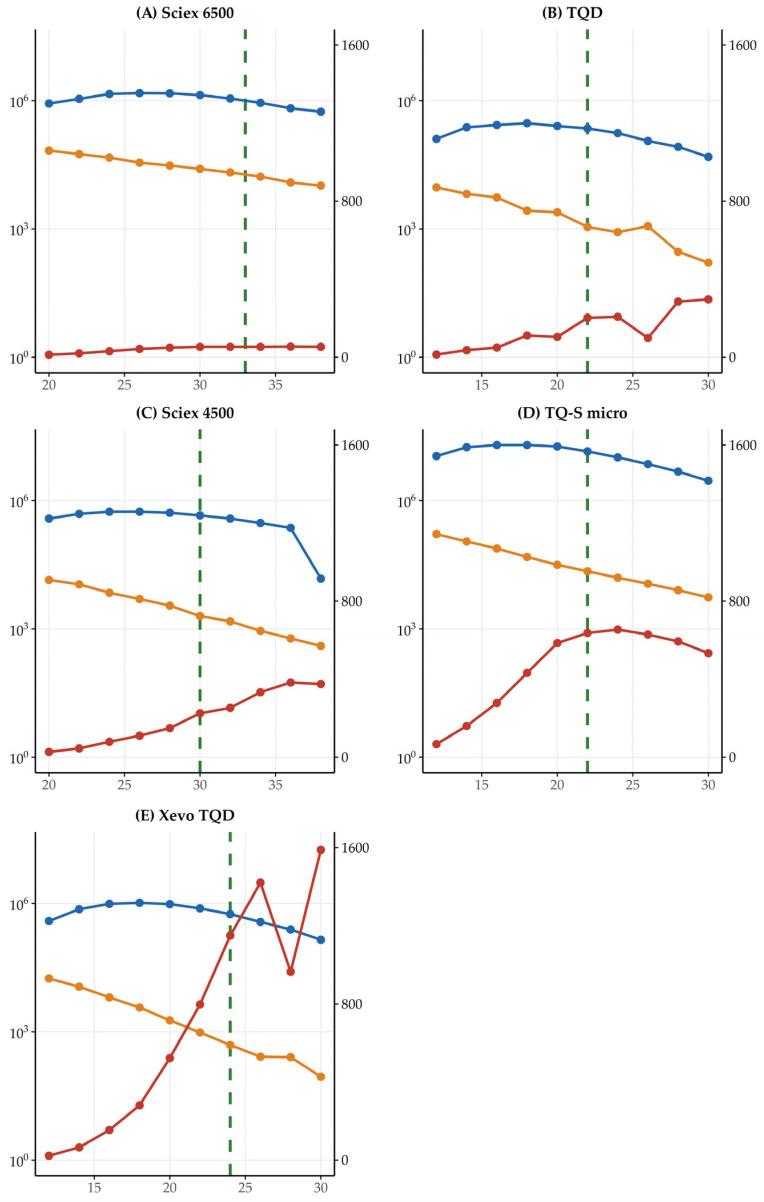
Illustration of de-tuning showing the relationship between signal intensity and collision energy for succinylacetone and methionine obtained whilst infusing a solution of succinylacetone SIL and methionine SIL (61 and 100 µmol/L respectively) on (**A**) Sciex 6500; (**B**) Waters TQD; (**C**) Sciex 4500; (**D**) Waters TQ-S micro; (**E**) Waters Xevo TQD. The signal intensity from 155.1 > 109.10 represents the contribution from the background signal attributable to the methionine SIL (+2 isotopic contribution due to ^34^S). The x-axis represents collision energy (eV). The primary y-axis (log scale) represents the signal intensity of the 160.1 > 109.1 and 155.1 > 109.1 transitions, represented in blue and orange respectively. The secondary y-axis represents the ratio of the signal intensities (160.1 > 109.1 to 155.1 > 109.1) which are shown in red. The dashed vertical green line indicate the optimized collision energy (eV) for each instrument.

De-tuning is achieved by direct infusion of a solution containing only the SILs into the MS. The MET SIL transition is optimized to achieve maximum signal intensity as normal, whereas the cone and collision energy (CE) for the SA SIL transition are adjusted to minimize the signal intensity recorded in the SA channel, i.e., that which is due to the MET SIL, whilst ensuring the signal from the SA SIL remains adequate. The sweet spot appears to be when the signal ratio of SA SIL: SA is >1000 which is typically achievable with a Waters Xevo TQD. However, on an older instrument such as a Waters TQD, this ratio is closer to 500, and on an AB Sciex API 6500, it is only around 50 because SA and MET show very similar CE vs intensity curves making it impossible to discriminate between them in this way (Figure 1A–E and Figure S1).

**Figure 2:**
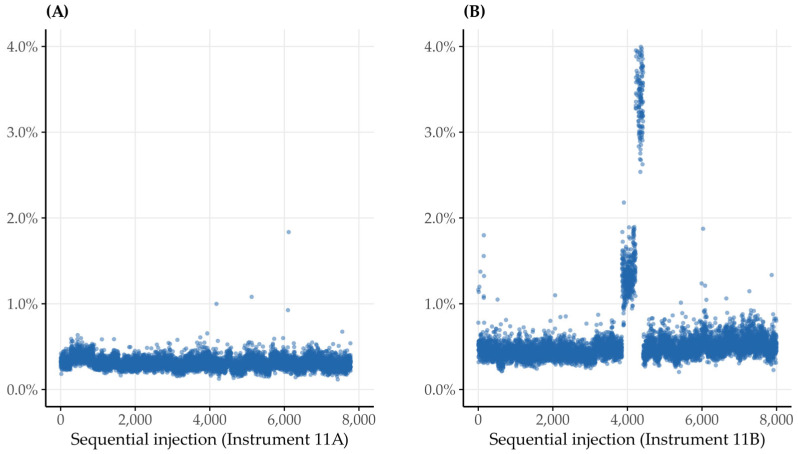
Percentage contribution (y axis) from methionine stable isotope label to 3-(5-methyl-1H-pyrazol-3-yl) propanoic acid (MPP), the marker analyte for endogenous succinylacetone, over sequential injections (x axis) of DBS specimens on two Waters Xevo TQDs: (**A**) Instrument 11A; (**B**) Instrument 11B.

Longitudinal monitoring of the signal intensity ratio for MET SIL to apparent endogenous SA on two Waters Xevo TQDs (11A and B) demonstrated that the magnitude of the background interference remains stable for a given instrument despite expected fluctuations in instrument performance (Figure 2). The only notable deviation occurred during a three-day period when the collision gas supply failed, during which the ratio gradually increased toward the theoretical 4% isotopic contribution. Regular monitoring of this ratio provides a pragmatic means of identifying instrument-specific limitations of the assay and differences in SA background interference between instruments, within and across laboratories.

### 3.3. Population Distributions and Evaluation of Screening Cut-Off Values

**Figure 3:**
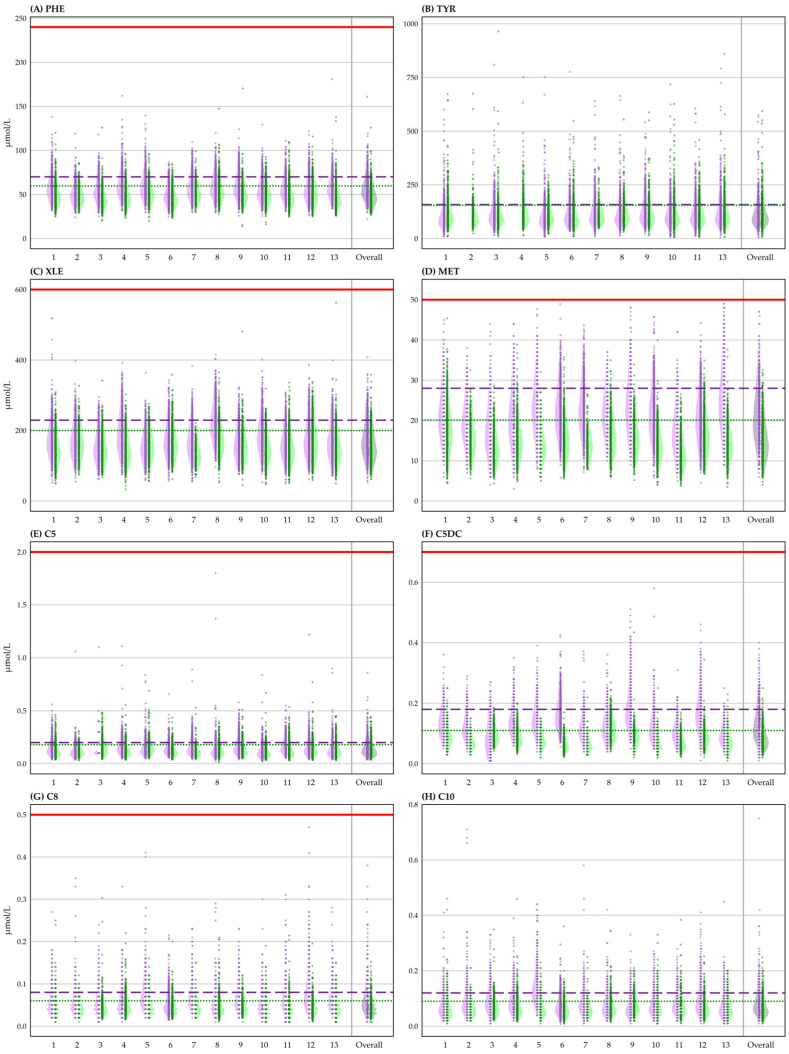
Comparison of LDT (purple) and NB2 (green) population distributions for (**A**) phenylalanine (PHE); (**B**) tyrosine (TYR); (**C**) leucine (LEU); (**D**) methionine (MET); (**E**) isovalerylcarnitine (C5); (**F**) glutarylcarnitine (C5DC); (**G**) octanoylcarnitine (C8); (**H**) decanoylcarnitine (C10) by laboratory. The dashed purple and dotted green horizontal lines represent the overall 90th centiles for LDT and NB2 respectively; the horizontal red line denotes the screening cut-off value (COV). Note that TYR and C10 are secondary markers; hence, no COV is denoted.

Implementation of NB2 reduced inter-laboratory variability for the existing eight analytes, excluding SA, compared with the LDTs, as demonstrated by between-laboratory dispersion ratios < 1.0 for the laboratory-level MADs, P90 and P99 centile distributions (Table 2). The exception was the ratio of between-laboratory SD of MADs for leucine. NB2 also showed reduced variability in extreme upper-tail behavior for all analytes except MET. This is considered consistent with the heterogeneity of MET in the neonatal population. Population summary statistics for both data sets are presented in Table 3 and summarized visually in Figure 3.

As expected, based on existing reports [18,19], NB2 demonstrated a negative bias relative to the LDT at both the median and P90 for all analytes. This ranged from −1.4% for tyrosine, to −39.6% for C5DC at the median, and −2.2% for tyrosine, to 40.3% for C5DC at P90 (Table 2). For the acylcarnitines, these comparisons are made at endogenous concentrations where imprecision is greatest, and the magnitude of the apparent bias therefore reflects both a true method difference and substantial analytical variability at the LLOQ. The negative bias raised the question of whether the existing LDT-derived COVs should be lowered proportionally for use with NB2. Following a review of the population data by a multi-disciplinary expert group including clinical and laboratory representatives, it was concluded that the existing COVs remain appropriate for the detection of PKU, MCADD, GA1, MSUD, HCU and IVA using NB2, on the basis that classical presentations of each disorder would continue to be reliably identified.

Determining the COV for SA required careful consideration of the instrument-specific background interference from the MET SIL, which effectively defines the LLOQ for each instrument. Review of the literature highlighted the wide range of COVs in use internationally and the impossibility of making meaningful comparisons across methods that are neither harmonized nor standardized. Kuypers et al. [19] reported a median SA COV of 1.5 µmol/L (range 0.3–7.0) across 31 screening programs; for those using NB2 specifically, COVs ranged from 0.3 to 2.0 µmol/L. Given the instrument-dependent background interference reported here (mean 0.22 µmol/L, range 0.05–0.62), adoption of the lower end of this range would provide little or no discrimination from background signal in the normal population, underscoring the inadequacy of adopting published COVs without reference to local analytical performance.

**Figure 4:**
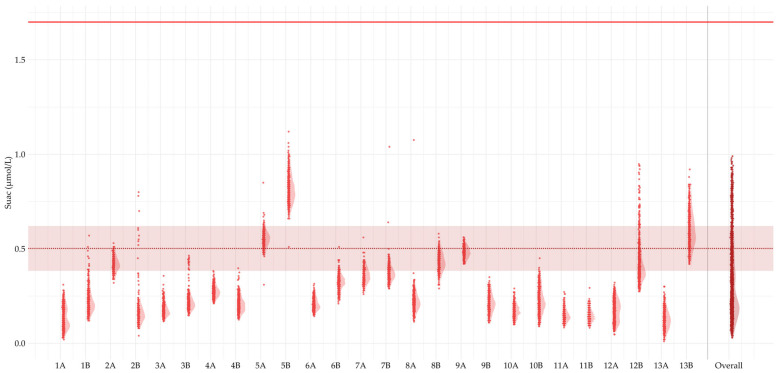
NB2 population distribution for succinylacetone by laboratory. The dotted horizontal line and the shaded band indicate the P90 for the overall data set and the MAD around the P90 respectively. The solid horizontal line denotes the screening COV.

The UK population data were therefore central to establishing the COV; however, it should be noted that standard confidence interval methods for population centiles could not be applied to SA as the data reflected the instrument specific interference, rather than the endogenous concentration of SA in healthy neonates. Across 26 instruments, the P99 for SA ranged from 0.21 to 0.99 µmol/L (Table 4 and Figure 4). Most instruments achieved P99 values < 0.6 µmol/L except for the 6A, 6B (Sciex 6500), 12B and 13A (Waters TQD), which had P99 of 0.63, 0.99, 0.80 and 0.82 µmol/L respectively (see Figure S2). These data were reviewed alongside reports from the literature. European data from seven countries using NB2, encompassing over 6.4 million screened infants, demonstrated an overall sensitivity of 98.5% and positive predictive value (PPV) of 56.0% [19]; programs using COVs ≥ 1.5 µmol/L achieved a higher mean PPV of 77.6% compared with 58.9% for those using COVs < 1.5 µmol/L, with no increase in false negatives. US laboratories using NB2 reported COVs ranging from 0.5 to 2.8 µmol/L (excluding one clear outlier), with a mean of 1.30 µmol/L. Taking the UK population data, international NB2 experience, and instrument-specific analytical performance together, the clinical COV was set at 1.7 µmol/L, and the analytical COV at 1.3 µmol/L, the latter accounting for intra-batch imprecision (12%) at the clinical COV. This was considered to offer acceptable sensitivity for the detection of classical HT1 while achieving a PPV of at least 50%.

## 4. Discussion

This study represents the first multi-center, national scale evaluation of NB2, including direct comparison of analytical performance and population distributions with historic LDTs across an integrated NBS program. The methodology change was driven by the dual requirements to implement screening for HT1 and to address longstanding challenges in harmonizing results from heterogeneous LDTs. Implementation of NB2 resulted in improved harmonization for the existing analytes, demonstrated by reduced between-laboratory dispersion of MAD and centile-based population metrics when compared with historic LDT data. These gains are important given the persistent inter-laboratory variability associated with LDT based screening methods both within and between screening programs. However, the observed reductions in inter-laboratory variability were not inherent to the commercial assay alone. Harmonization was achieved only after application of instrument-specific RRFs to all analytes. That 13% of RRFs adjusted measured concentrations by more than 20% is striking and appears inconsistent with the principles of SID-MS, in which stable isotopes labeled IS are expected to compensate for variability arising from extraction efficiency, ionization efficiency, and matrix effects.

This finding underscores that while harmonization can nominally be achieved through post-analytical correction factors, this does not represent analytical standardization and raises important questions regarding traceability. The application of analyte-specific RRFs in newborn screening therefore warrants careful consideration. In practice, this approach represents a single-point calibration performed at a fixed time using materials with analyte concentrations significantly higher than the region of analytical interest, the COV. Applying RRFs that are smaller than the inherent analytical imprecision of an analyte may be counter-intuitive and of questionable analytical value. For example, RRFs applied to C8 ranged from 0.88 to 1.09 while the mean imprecision was ±9.3%. In contrast, RRFs for C5DC ranged from 0.77 to 1.55, far greater than the mean imprecision of ±12.8%, suggesting a more fundamental measurement discrepancy. Whilst it is acknowledged that analytical performance specifications for screening differ from those of diagnosis or monitoring, laboratories nonetheless have a responsibility to ensure that results are consistent, comparable and fit for purpose [23]. As quantitation around the COV determines whether or not a baby is referred for follow up, ensuring a high degree of confidence in measurement accuracy and comparability at this decision threshold is key. This work further highlights the need for internationally available, matrix-matched certified reference materials to support meaningful standardization of newborn screening assays.

SA measurement remains a significant analytical limitation of the NB2 assay, the impact of which is instrument dependent. Background interference arising from the MET SIL ranged from approximately 0.2 μmol/L on Waters Xevo TQD instruments to 0.7 μmol/L on Sciex 6500 platforms. These differences reflect a fundamental overlap in the collision-induced fragmentation behavior of SA, which cannot be fully mitigated by Manufacturer-applied de-tuning strategies. Consequently, laboratories must establish instrument-specific LLOQs and ensure that SA COVs are set with reference to local analytical performance rather than published values alone. This observation aligns with international reports demonstrating wide variation in SA COVs across programs using different methodologies and instrumentation. While background interference is unlikely to compromise detection of classical HT1, it raises questions about sensitivity for atypical presentations with milder biochemical profiles.

Recovery was acceptable for most analytes; however, two findings merit specific comment. First, SA recovery was consistently low (mean 53%, range 31–72%), a previously reported limitation of both derivatized and non-derivatized MS/MS methods. Second, C10 showed consistent over-recovery (mean 112%, range 97–130%), with most instruments exceeding 110% recovery. Although such biases are unlikely to affect binary screening decisions which are based on both increased C8 and increased C8:C10 ratio, they highlight the absence of traceable calibration hierarchies.

A fundamental implication of this work is that triple quadrupole MS are not analytically interchangeable. Differences in ion source design and transmission, collision cell architecture and vacuum characteristics can result in instrument specific ion energies, fragmentation patterns and transition behaviors which can have practical consequences, particularly in FIA-MS/MS methods where chromatographic selectivity is absent. As NBS panels continue to expand and analytical complexity increases, reliance on dilute-and-shoot approaches without calibration may no longer be adequate. While FIA-MS/MS remains operationally efficient and clinically effective, greater attention to instrument-specific behavior, interferences, and traceability will be required to ensure long-term reliability and comparability of screening outcomes.

## 5. Conclusions

This study demonstrates that implementation of a single commercial assay across a national NBS program can improve harmonization, with reduced inter-laboratory variation observed for all analytes relative to historic LDTs. However, these gains were only achievable through the application of significant instrument-specific correction factors, highlighting limitations in analytical standardization and raising important concerns regarding traceability. Analytical challenges associated with SA, including instrument-dependent background interference, underline the necessity for laboratories to establish instrument-specific limits of quantitation and evidence-based, population-specific screening COV rather than adopting published thresholds without reference to local analytical performance. Taken together, these findings demonstrate progress toward harmonized screening delivery in England, while emphasizing that harmonization achieved through correction factors does not equate to standardization. At the level of individual analytes, fitness for purpose is not conferred by commercial kit adoption alone, but requires laboratories to understand the specific performance characteristics of their assay and instrument—a consideration particularly critical for SA, where the COV must account for instrument-dependent background interference rather than being extrapolated from programs using different methods or platforms [23]. The work highlights the international need for traceable, matrix-matched reference materials and robust calibration hierarchies to support consistent, comparable screening outcomes across laboratories, instruments, and screening programs worldwide.

## Figures and Tables

**Table 1:** All laboratory mean imprecision by analyte determined from replicate measurements (*n* = 25) of the three third-party internal quality control (IQC) materials.

Analyte	All Lab Mean (µmol/L)	Mean Intra-Lab Imprecision (%CV)	All Lab SD	Inter-Laboratory Imprecision (%CV)
Phenylalanine	76	8.8	4.2	5.5
	146	7.6	8.6	5.9
	294	8.7	15.4	5.2
Tyrosine	70	8.2	3.7	5.3
	258	7.1	12.5	4.9
	523	7.8	22.6	4.3
Leucine	218	9.6	8.5	3.9
	303	8.7	13.8	4.6
	511	10.1	22.3	4.4
Methionine	23	9.8	0.9	3.8
	56	9.0	2.5	4.5
	178	10.0	6.8	3.8
C5 carnitine	0.2	10.5	0.02	10.2
	0.6	8.9	0.1	10.1
	2.3	9.7	0.2	8.8
C5DC carnitine	0.1	12.1	0.01	18.8
	0.3	9.5	0.04	14.4
	1.4	9.7	0.2	14.9
C8 carnitine	0.2	10.4	0.01	8.7
	0.6	8.5	0.1	8.2
	2.3	9.1	0.2	6.7
C10 carnitine	0.2	10.8	0.01	9.1
	0.5	9.9	0.04	8.6
	2.1	11.1	0.2	7.4
Succinylacetone	0.41	20.8	0.09	20.8
	2.3	11.6	0.2	9.6
	5.3	12.2	0.4	8.3

**Table 2:** Comparator statistics for Neobase™ 2 (NB2) (*n* = 23,791) vs. laboratory-developed tests (LDT) (*n* = 139,444) for the existing analytes.

	% Bias to LDT		Ratio of Between-Laboratory Standard Deviations			
Analyte (µmol/L)	Median	P90	MAD	P90	P99	Period Centered P99
Phenylalanine	−15.2	−15.1	0.80	0.80	0.89	0.22
Tyrosine	−1.4	−2.2	0.78	0.62	0.91	0.19
Leucine	−12.2	−12.1	1.11	0.75	0.84	0.97
Methionine	−30.0	−29.2	0.88	0.74	0.84	1.14
C5 carnitine	−13.2	−14.6	0.47	0.85	0.94	0.67
C5DC carnitine	−39.6	−40.3	0.81	0.48	0.40	0.66
C8 carnitine	−28.4	−28.8	0.33	0.27	0.32	0.28
C10 carnitine	−24.0	−24.0	0.43	0.30	0.51	0.34

**Table 3:** Summary of population data obtained using the LDTs (*n* = 139,444) and NB2 (*n* = 23,791), the latter denoted by grey shading.

Analyte (µmol/L)	P1	P10	Median	Mean	MAD	P90	95% CI P90	P99	Separation Margin % (p99)
Phenylalanine	36.8	44.1	54.6	55.9	6.2	69.2	68.5–69.6	88.8	63.1
	31.2	37.4	46.3	47.5	5.3	58.7	57.5–60.2	75.3	68.6
Tyrosine	49.6	62.8	96.1	106.1	22.1	157.1	154.1–159.9	285.5	-
	43.1	62.5	94.8	104.0	21.6	153.6	146.3–161.6	266.1	-
Leucine	96.9	126.1	167.7	172.1	24.9	224.2	222.2–226.2	246.5	58.9
	84.5	110.2	147.2	151.1	22.2	197.1	192.1–201.9	250.0	58.3
Methionine	10.9	14.9	20.4	20.9	3.3	27.4	27.2–27.6	35.0	30.0
	7.8	10.4	14.3	14.7	2.3	19.4	18.9–19.9	25.0	50.0
C5 carnitine	0.06	0.08	0.13	0.14	0.03	0.20	0.20–0.21	0.33	83.5
	0.05	0.07	0.11	0.12	0.02	0.17	0.17–0.18	0.28	86.0
C5DC carnitine	0.07	0.09	0.12	0.13	0.02	0.17	0.17–0.17	0.24	65.7
	0.04	0.06	0.07	0.08	0.02	0.10	0.10–0.11	0.14	80.0
C8 carnitine	0.02	0.04	0.05	0.06	0.01	0.08	0.08–0.08	0.14	72.0
	0.02	0.03	0.04	0.04	0.01	0.06	0.05–0.06	0.09	82.0
C10 carnitine	0.03	0.05	0.07	0.08	0.02	0.12	0.11–0.12	0.20	-
	0.02	0.04	0.06	0.06	0.01	0.09	0.09–0.10	0.15	-
Succinylacetone	-	-	-	-	-	-	-	-	-
	0.20	0.23	0.27	0.27	0.02	0.33	0.32–0.33	0.40	76.2

**Table 4:** Summary of succinylacetone population data by instrument.

Instrument (*n*)	P1	P10	Mean	Median	MAD	P90	95% CI P90	P99	Separation Margin (%)
1A (1689)	0.05	0.07	0.13	0.11	0.03	0.20	0.20–0.20	0.25	85.3
1B (1045)	0.14	0.16	0.21	0.20	0.02	0.27	0.26–0.28	0.39	77.1
2A (594)	0.34	0.37	0.42	0.41	0.02	0.46	0.45–0.47	0.50	70.6
2B (437)	0.08	0.11	0.17	0.15	0.02	0.20	0.20–0.22	0.59	65.3
3A (699)	0.13	0.14	0.17	0.17	0.02	0.21	0.20–0.21	0.26	84.5
3B (649)	0.16	0.18	0.21	0.21	0.02	0.25	0.24–0.26	0.43	74.6
4A (968)	0.22	0.24	0.27	0.27	0.02	0.30	0.30–0.31	0.34	80.2
4B (1071)	0.14	0.16	0.20	0.20	0.02	0.25	0.24–0.25	0.29	83.2
5A (681)	0.48	0.51	0.55	0.55	0.02	0.59	0.59–0.60	0.63	62.8
5B (829)	0.69	0.74	0.81	0.80	0.04	0.90	0.89–0.90	0.99	41.8
6A (932)	0.16	0.17	0.20	0.20	0.02	0.24	0.24–0.24	0.27	84.2
6B (1058)	0.24	0.29	0.33	0.33	0.02	0.36	0.36–0.36	0.40	76.5
7A (516)	0.28	0.32	0.36	0.36	0.02	0.40	0.40–0.41	0.44	74.1
7B (999)	0.31	0.33	0.37	0.36	0.02	0.40	0.40–0.41	0.45	73.5
8A (1012)	0.14	0.17	0.22	0.21	0.02	0.26	0.26–0.27	0.30	82.1
8B (434)	0.31	0.37	0.43	0.42	0.03	0.48	0.48–0.49	0.53	68.8
9A (540)	0.42	0.45	0.48	0.48	0.02	0.52	0.51–0.52	0.55	67.8
9B (630)	0.12	0.16	0.21	0.21	0.03	0.27	0.26–0.28	0.31	81.8
10A (817)	0.11	0.13	0.17	0.17	0.02	0.21	0.20–0.21	0.25	85.4
10B (474)	0.11	0.16	0.22	0.21	0.03	0.30	0.29–0.30	0.36	78.8
11A (821)	0.09	0.11	0.14	0.14	0.01	0.17	0.17–0.18	0.22	87.1
11B (1223)	0.09	0.12	0.14	0.14	0.01	0.16	0.16–0.18	0.21	87.7
12A (1391)	0.07	0.10	0.17	0.17	0.04	0.24	0.23–0.24	0.28	83.7
12B (1309)	0.30	0.34	0.40	0.38	0.03	0.49	0.47–0.49	0.80	53.2
13A (753)	0.44	0.50	0.60	0.59	0.06	0.71	0.69–0.72	0.82	51.8
13B (1220)	0.04	0.07	0.12	0.12	0.03	0.18	0.17–0.18	0.24	85.9

## Data Availability

The data that support the findings of this study are available from NHS England. Restrictions apply to the availability of these data. Data are available from the corresponding author (RSC) with the permission of NHS England.

## Supplementary Materials

The following supporting information can be downloaded at https://www.mdpi.com/article/10.3390/ijns12020041/s1. Supplementary file S1. Table S1: Optimized analyte relative response factors (RRF) for each instrument displayed as the factor applied to the final measured result. Table S2: All laboratory mean recovery for each analyte relative to the enriched concentration of the Revvity multi-level DBS materials L1 to L6. Figure S1: 3D plot showing the effect of collision energy on the signal intensity of the succinylacetone SIL (160.1 > 109.10) and the +2 (^34^S) isotope of the methionine SIL (155.1 > 109.10) during direct infusion of internal standard mixture on a Waters Xevo TQD (red) and a Sciex 6500 (blue). The dotted line indicates the optimised collision energy for each instrument. On the Xevo TQD, maximum signal intensity of the two transitions occurs at different collision energies, enabling discrimination of the interference by collision energy optimisation. On the Sciex 6500, maximum intensity for both transitions occurs at very similar collision energies, precluding effective discrimination. Table S3: Inter-lot variation for each analyte across all thirteen laboratories for the three third party IQC materials. Lot A and B refer to internal standard kit lots 759382 and 766157 respectively. Figure S2: Comparison of succinylacetone population data by instrument type, and for all 13 laboratories. The dashed line and the shaded band represent the P90 and the MAD around the P90 for all laboratories. The solid line represents the clinical cut-off value. Concentration in µmol/L.

## Abbreviations

The following abbreviations are used in this manuscript:

COV	Cut-off value
CV	Coefficient of variation
C10	Decanoylcarnitine
DBS	Dried blood spot
CE	European conformity
FIA-MS/MS	Flow injection analysis tandem mass spectrometry
GA1	Glutaric aciduria type 1
C5DC	Glutarylcarnitine
HT1	Hereditary tyrosinemia type 1
IQC	Internal quality control
IS	Internal standard
IVDR	In Vitro Diagnostic Regulation (EU) 2017/746
IVA	Isovaleric acidemia
C5	Isovalerylcarnitine
LDT	Laboratory-developed test
LEU	Leucine
LLOQ	Lower limit of quantitation
MSUD	Maple syrup urine disease
MS	Mass spectrometer
MAD	Median absolute deviation
MCADD	Medium-chain Acyl-CoA dehydrogenase deficiency
MET	Methionine
MHRA	Medicines & Healthcare products Regulatory Agency
MPP	3-(5-methyl-1H-pyrazol-3-yl) propanoic acid
NB2	Neobase™ 2
NBS	Newborn screening
NSQAP	Newborn Screening Quality Assurance Program
C8	Octanoylcarnitine
PHE	Phenylalanine
PKU	Phenylketonuria
PPV	Positive predictive value
HCU	Pyridoxine unresponsive homocystinuria
RRF	Relative response factor
SIL	Stable isotope label
SD	Standard deviation
SA	Succinylacetone
SID-MS	Stable isotope dilution mass spectrometry
TYR	Tyrosine
