# Supplementary material for: Transitioning from Laboratory-Developed Tests to a Single Commercial Reagent Kit in a National Newborn Screening Program: Impact on Analytical Performance and Harmonization
Source: Int J Neonatal Screen. 2026 Jun 9;12(2):41. doi: 10.3390/ijns12020041 (PMC13300528; doi:10.3390/ijns12020041)

Table S1. Optimized analyte relative response factors (RRF) for each instrument displayed as the factor applied to the final measured result. [1]

Instrument	ID	Phe	Tyr	Leu	Met	C5	C5DC	C8	C10	SA
TQD	10A	1.11	1.17	0.93	0.90	1.13	0.98	1.05	0.98	1.01
TQD	10B	1.06	1.10	0.91	0.93	0.95	0.94	0.99	1.27	1.13
TQD	12A	1.16	*	1.08	0.93	1.06	1.07	1.07	1.26	0.77
TQD	13A	0.96	1.14	0.86	0.83	0.92	0.95	0.97	1.28	0.80
Xevo TQD	11A	0.98	0.98	0.82	1.19	1.03	1.02	0.95	1.06	1.21
Xevo TQD	11B	1.07	1.03	0.99	1.04	0.98	0.93	1.02	1.19	1.22
Xevo TQD	12B	1.04	*	1.06	0.94	1.14	1.07	1.05	1.04	0.74
Xevo TQD	13B	0.96	0.94	0.93	0.95	0.89	0.96	0.97	1.25	0.93
Xevo TQD	1A	0.93	0.93	0.85	1.06	0.94	1.00	0.96	1.07	0.92
Xevo TQD	1B	0.99	0.98	1.01	1.10	0.96	1.00	1.05	1.17	0.94
Xevo TQD	2B	1.03	1.08	1.00	1.19	0.95	1.00	1.01	1.20	1.21
Xevo TQD	8A	0.92	1.02	0.88	0.93	0.88	1.05	1.00	1.02	1.34
Xevo TQSμ	2A	1.19	1.11	1.15	1.03	1.02	1.05	1.09	1.39	1.15
Xevo TQSμ	6A	1.03	1.10	1.06	1.04	0.90	0.89	1.01	1.46	0.93
Xevo TQSμ	6B	0.99	1.04	0.92	0.99	0.91	0.92	0.97	1.61	0.87
Xevo TQSμ	7A	1.19	1.19	1.16	1.13	1.18	1.55	1.07	1.01	1.15
Xevo TQSμ	7B	1.26	1.28	1.14	1.16	1.03	1.05	1.02	1.24	1.12
Sciex 4500	3A	0.94	0.97	1.03	0.89	0.85	0.77	0.98	1.27	0.97
Sciex 4500	4A	0.82	0.98	1.01	1.02	0.88	0.93	1.02	1.10	0.72
Sciex 4500	4B	1.02	0.88	1.33	1.06	0.92	0.82	1.03	1.08	0.87
Sciex 5000	9B	0.96	1.05	1.27	1.10	1.05	0.93	1.03	1.28	1.16
Sciex 5500	3B	1.06	1.11	0.93	0.99	1.03	0.85	1.05	1.20	0.90
Sciex 6500	5A	1.08	1.10	1.04	1.10	0.94	0.93	1.01	1.30	0.93
Sciex 6500	5B	1.12	1.11	1.03	1.32	0.89	0.88	1.02	1.28	1.06
Shimadzu 8050	8B	1.02	1.01	0.90	0.97	0.85	0.84	0.88	1.18	0.99
Shimadzu 8050	9A	1.10	1.17	1.19	0.97	0.81	0.97	1.08	1.39	0.87
Mean		1.04	1.06	1.02	1.03	0.87	0.98	1.01	1.21	1.00
Min		0.82	0.88	0.82	0.83	0.81	0.77	0.88	0.98	0.72
Max		1.26	1.28	1.33	1.32	1.18	1.55	1.09	1.61	1.34

[1] RRF are displayed as the factor applied to the final measured concentration. This is to account for differences in the instrument software.

Table S2. All laboratory mean recovery for each analyte relative to the enriched concentration of the Revvity multi-level DBS materials L1 to L6.

Instrument	Phe	Tyr	Leu	Met	C5	C5DC	C8	C10	SA
Mean recovery (%)	87	89	82	88	83	85	94	112	53
Min	75	72	72	75	68	81	54	97	31
Max	101	100	92	97	97	113	121	130	72

Figure S1: 3D plot showing the effect of collision energy on the signal intensity of the succinylacetone SIL (160.1 > 109.10) and the +2 (^{34}S) isotope of the methionine SIL (155.1 > 109.10) during direct infusion of internal standard mixture on a Waters Xevo TQD (red) and a Sciex 6500 (blue). The dotted line indicates the optimised collision energy for each instrument. On the Xevo TQD, maximum signal intensity of the two transitions occurs at different collision energies, enabling discrimination of the interference by collision energy optimisation. On the Sciex 6500, maximum intensity for both transitions occurs at very similar collision energies, precluding effective discrimination.

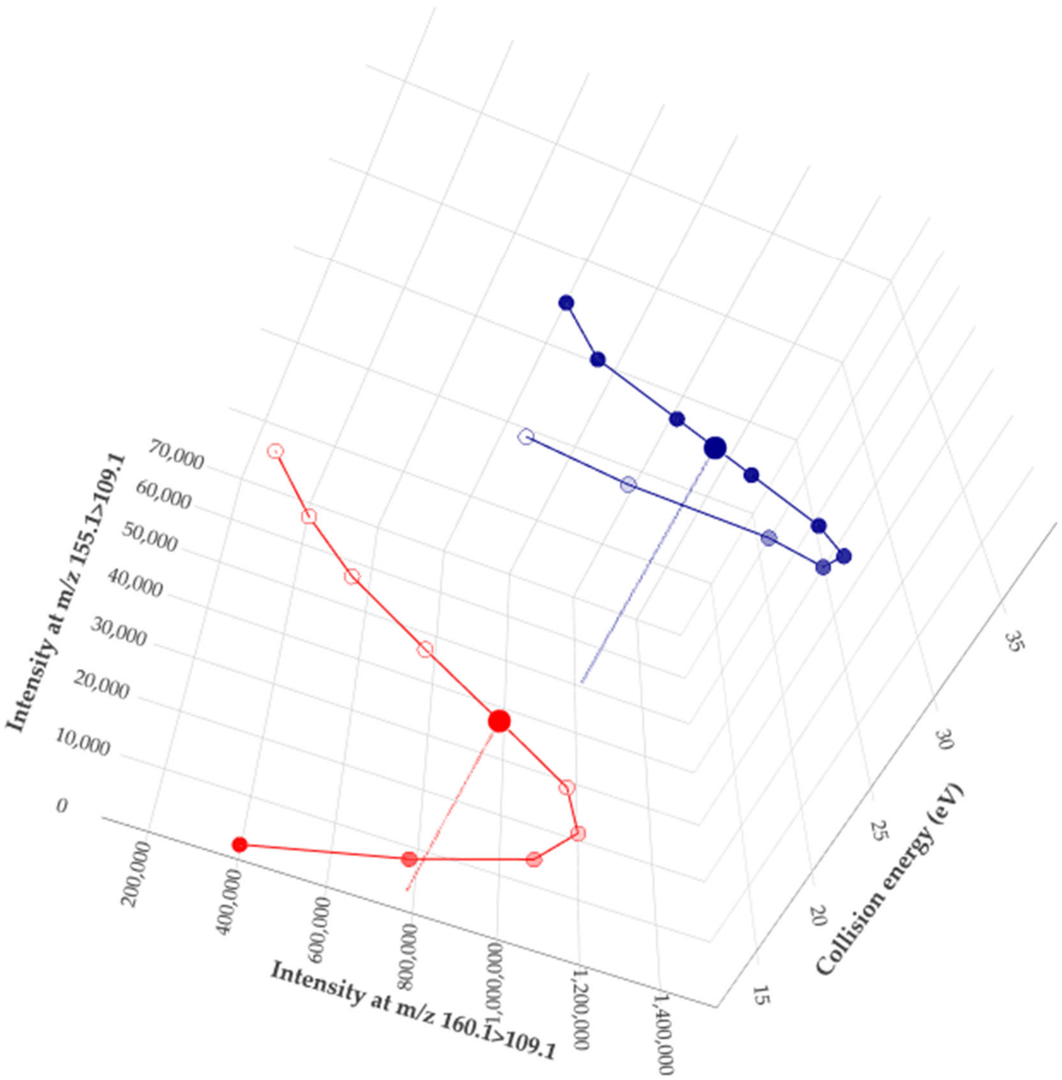

Table S3: Inter-lot variation for each analyte across all thirteen laboratories for the three third party IQC materials. Lot A and B refer to internal standard kit lots 759382 and 766157 respectively.

Material	Phe	Tyr	Leu	Met	C5	C5DC	C8	C10	SA
**IQC Level 1**									
Lot A Mean concentration (µM)	76.8	70.0	217.3	22.6	0.2	0.11	0.15	0.15	0.41
Lot B Mean concentration (µM)	77.2	70.3	215.1	22.8	0.2	0.10	0.15	0.15	0.42
Bias between lots (%)	0.6	0.4	-0.8	0.8	1.2	-2.3	0.7	3.3	5.3
Relative difference (%) between lots	0.4	0.1	-0.9	0.6	1.0	-2.6	0.5	2.9	4.7
Inter lot %CV	8.3	6.9	5.6	11.1	13.3	13.8	10.1	11.5	35.2
**IQC Level 2**									
Lot A Mean concentration (µM)	145.4	255.9	298.4	56.4	0.6	0.29	0.60	0.49	2.48
Lot B Mean concentration (µM)	146.8	257.7	297.1	56.3	0.6	0.28	0.59	0.49	2.51
Bias between Lots (%)	1.1	0.7	-0.4	-0.1	0.0	-2.5	-0.8	1.9	2.0
Relative difference (%) between lots	1.0	0.6	-0.4	-0.2	-0.1	-2.8	-0.9	1.8	1.6
Inter lot %CV	6.9	5.6	5.4	10.9	11.9	11.9	7.7	13.6	18.0
**IQC Level 3**									
Lot A Mean concentration (µM)	297.1	523.0	508.0	180.2	2.4	1.44	2.29	2.03	5.77
Lot B Mean concentration (µM)	301.5	531.7	511.0	181.5	2.4	1.43	2.29	2.10	5.78
Bias between Lots (%)	1.5	1.7	0.7	0.9	1.0	-0.4	0.1	3.2	0.5
Relative difference (%) between lots	1.5	1.6	0.6	0.8	0.9	-0.5	0.0	3.0	0.1
Inter lot %CV	8.1	6.2	6.5	12.0	13.3	10.1	9.4	12.4	22.6
**Mean inter lot %CV**	**7.8**	**6.2**	**5.8**	**11.4**	**12.8**	**11.9**	**9.1**	**12.5**	**25.3**
**Mean bias between lots (%)**	1.1	0.9	-0.2	0.5	0.8	-1.7	0.0	2.8	2.6
**Mean relative difference between lots (%)**	1.0	0.8	-0.3	0.4	0.6	-1.9	-0.1	2.6	2.1

Figure S2: Comparison of succinylacetone population data by instrument type, and for all 13 laboratories. The dashed line and the shaded band represent the P90 and the MAD around the P90 for all laboratories. The solid line represents the clinical cut-off value. Concentration in µmol/L.

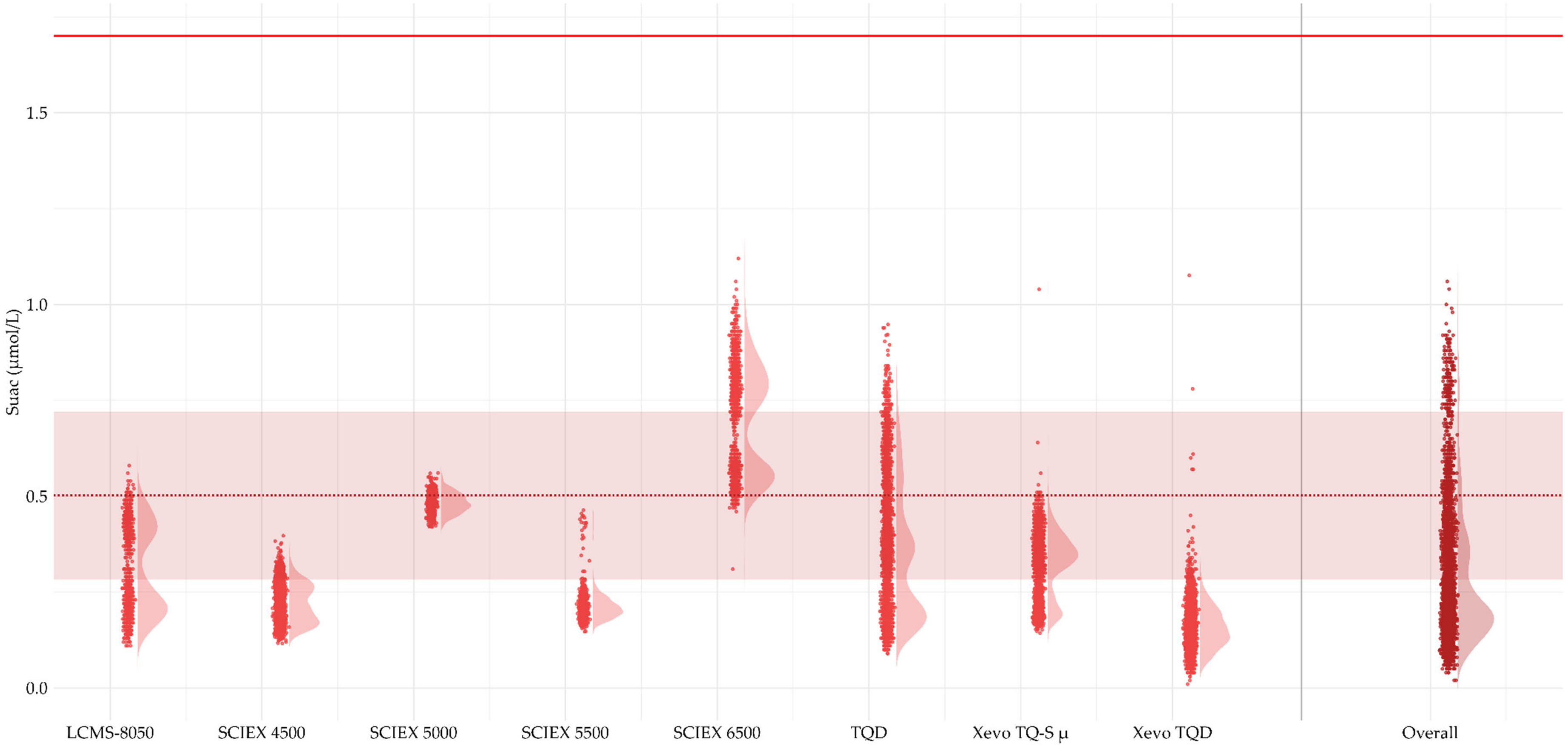

Supplement: Supplementary file 1 [file IJNS-12-00041-s001.zip › IJNS-4324541-supplementary.pdf]
